# Defining conditions for biofilm inhibition and eradication assays for Gram-positive clinical reference strains

**DOI:** 10.1186/s12866-018-1321-6

**Published:** 2018-11-03

**Authors:** Cristina D. Cruz, Shreya Shah, Päivi Tammela

**Affiliations:** Drug Research Program, Division of Pharmaceutical Biosciences, Faculty of Pharmacy, FI-00014 University of Helsinki, P.O. Box 56, Helsinki, Finland

**Keywords:** Biofilm, Media, Glucose, Gram-positive bacteria, Crystal violet, Resazurin, Ciprofloxacin, Linezolid, MBIC, MBEC

## Abstract

**Background:**

Biofilms are formed by a complex bacterial community encapsulated by a polymeric matrix, with strong adherent properties and persistent phenotype. Biofilms are considered one of the most challenging areas of modern medicine. Existing antibiotics have been developed against free-floating bacterial cells, and thus, many treatments of biofilm-related infection fail. In this study, we compared the effects of different media on biofilm growth of clinical reference strains of *Staphylococci* and *Enterococci*, including multi-drug resistant representatives. Further, we optimized the resazurin-based assay for determining the minimal biofilm inhibitory concentration (MBIC) of standard antibiotics, and evaluated its use for the determination of minimal biofilm eradication concentration (MBEC).

**Results:**

We showed that tryptic soy broth supplemented with 1% glucose was an optimal media for maximum biofilm growth of all strains tested, with an extended incubation time for *Enterococci.* A range of parameters were tested for the resazurin assay, including concentration, temperature and time of incubation. Using quality parameters to analyze the assay’s performance, the conditions for the resazurin assay were set as follows: 4 μg/mL and 8 μg/mL, with incubation at 25 °C for 20 min and 40 min for *Staphylococci and Enterococci*, respectively.

**Conclusions:**

In summary, we defined conditions for optimal biofilm growth and for standardized resazurin assay for MBIC determination against six Gram-positive clinical reference strains. We also observed that MBEC determination by the resazurin-based assay is limited due to the poor detection limit of the assay. Complementary cell counting data is needed for precise determination of MBEC.

**Electronic supplementary material:**

The online version of this article (10.1186/s12866-018-1321-6) contains supplementary material, which is available to authorized users.

## Background

Biofilms can be described as a structured community of bacterial cells enclosed in a polymeric matrix and adherent to an abiotic or biotic surface. Bacteria account for 5–35% of biofilm volume. The remaining volume is the extracellular matrix, which is an enclosed, hydrated polyanionic complex of exopolysaccharide (EPS) [1]. In contrast to planktonic bacteria, biofilms provide a survival advantage to the microbial community, showing a nearly 1000-fold increase in antimicrobial tolerance [2].

In recent years, there has been an increase in the number of patients in health care receiving implanted biomaterials, which are notably prone to biofilm colonization [3–5]. Additionally, biofilms play a role in non-device-associated chronic bacterial infections [1, 6]. Treatment of these infections is not always successful due to bacterial tolerance to conventional antimicrobial agents, thus frequently leading to the surgical removal of the implanted device, involving risks and complications. In this context, biofilm-related infections caused by Gram-positive *cocci* are well established. For example, *Staphylococci is* one of the leading causative bacteria of catheters and prosthetic related-infections [4], followed by *Enterococci* [6, 7].

In general, for routine quantification of bacterial biofilms, total biomass measurements are used based on crystal violet staining, which stains both living and dead cells. Another method commonly used is based on resazurin, a blue non-fluorescent redox dye that is reduced by cellular metabolic activity to highly fluorescent, pink resorufin [8, 9]. Developments in the field of new antimicrobial agents do not take into account the characteristics of bacteria as biofilms [10], thus the ongoing need for effective biofilm treatment requires standardized screening methods for the reference laboratory. Currently, there is no gold standard method for assessing new anti-biofilm drugs.

Therefore, simple and standardized guidelines for optimal in vitro biofilm production and anti-biofilm susceptibility assays for Gram-positive laboratory reference strains are needed. To our knowledge, this is the first report in which the MBIC and MBEC assay set-ups for clinical reference strains of *Staphylococci* and *Enterococci* are systematically assessed and optimized by using the following assay quality parameters, commonly used in high-throughput screening: Z prime (Z’), signal to background (S/B) and signal window (SW). SW and Z’ are calculations that measure the fold response between maximum and minimum control signals and the precision of this response (variability). Z’ is a representation of SW using a score ranging from 0 to 1 and it is more reliable on assessing assay’s acceptability in comparison to SW. S/B is calculated taking into account the averages of minimum and maximum signals only. Acceptable values for the three parameters are: S/B and SW > 2-fold and for Z’ > 0.5 [11].

In particular, this study aimed to 1) evaluate the effects of different media on biofilm production, 2) establish optimal resazurin assay conditions and 3) assess the minimal biofilm inhibitory and eradication concentrations (MBIC and MBEC, respectively) for ciprofloxacin and linezolid against the clinical reference strains using the optimized methodology.

## Methods

### Bacterial strains

Six bacterial strains (Table 1) were purchased from Microbiologics Inc. (St. Cloud, MN, USA), and reconstituted as per the manufacturer’s instructions. Bacterial stocks were prepared in cation-adjusted Mueller Hinton broth (MHB, Becton Dickinson, Franklin Lakes, NJ, USA) and stored at − 80 °C. Fresh cultures were initiated on Mueller Hinton agar (MHA) plates on a monthly basis. Overnight cultures were prepared before the assay by subculturing bacterial strains on fresh MHA plates and incubated at 37 °C for 16–20 h.

**Table 1:** List of bacterial strains used in this study and the corresponding antibiotic profiles

Bacterial strain	ATCC number	Antibiotic profile
*Staphylococcus aureus*	29213	No resistance found
Methicillin-resistant *Staphylococcus aureus*	43300	Resistant to methicillin, oxacillin
*Enterococcus faecium*	35667	No resistance found
Vancomycin-resistant *Enterococcus faecium*	70021	Resistant to vancomycin, teichoplanin
*Enterococcus faecalis*	29212	No resistance found
Vancomycin-resistant *Enterococcus faecalis*	51575	Resistant to gentamicin, streptomycin, vancomycin

### Biofilm production

A single colony was taken from the MHA overnight bacterial culture, inoculated into 0.85% saline solution and vortexed to ensure that the bacterial suspension was homogeneous. Bacterial suspensions were analysed using a densitometer (DEN-1, BioSan, Warren MI, USA) and adjusted to 1 × 10^6^ colony forming units (CFU/mL) by diluting with appropriate broth. The broths used were MHB, Tryptic Soy (TS, BD), Tryptic Soy supplemented with 1% glucose (TSG, ICN Biomedicals, Irvine, CA, USA), or 2% glucose (TS2G), Brain Heart Infusion (BHI, Sigma-Aldrich, St Louis, MO, USA) and Brain Heart Infusion supplemented with 1% glucose (BHIG). An aliquot of 200 μL of bacterial suspension per well was dispensed into a 96-well flat bottom microplate (Nunc, Roskilde, Denmark). Negative control wells were filled with 200 μL of media only. Microplates were then incubated at 37 °C for 24 h [*Staphylococcus aureus* and methicillin-resistant *S. aureus* (MRSA)] or 48 h [*Enterococcus faecalis*, vancomycin- resistant *E. faecalis* (VRE), *Enterococcus faecium* and *E. faecium* VRE].

### Assessment of biofilm viable cells by colony count

Media was removed from all wells after the respective incubation times. The formed biofilm was washed once with 200 μL of phosphate-buffered saline (PBS). Next, 100 μL of PBS solution was added to wells containing biofilm and then biofilm cells were suspended by vigorous pipetting. The suspended biofilm was transferred to a new 96-well flat bottom microplate followed by 10-fold dilutions prepared in PBS. Five drops of 10 μL each was drop-plated on the agar respective to the broths used for biofilm production (e.g. MHA for biofilm grown in MHB). CFU were enumerated after 24 h of incubation at 37 °C. The experiment was performed twice with three replicates.

### Assessment of biofilm biomass by crystal violet staining

Biofilm biomass measurements by crystal violet (CV) staining were performed as previously described [12] with some modifications. An aliquot of 190 μL of 0.01% CV (Sigma-Aldrich) aqueous solution was added to three wells of the 96-well flat bottom microplate containing biofilm, along with its respective control media (three wells), and incubated at room temperature for 30 min. Then, CV solution was removed and wells were washed three times with 200 μL of sterile water. During this wash step care was taken not to disturb the biofilm. The plate was left to dry for 30 min at 50 °C. Next, 200 μL of 96–99% ethanol was added to each well and biofilm was detached by vigorous pipetting. Absorbance measurement values at 570 nm were obtained using the Multiskan GO (Thermo Fisher Scientific, Vantaa, Finland). If a negative value for optical density (OD) was obtained, it was presented as zero. The experiment was performed twice with three replicates.

### Assessment of metabolic activity of biofilm cells by resazurin

Biofilm production was performed using TSG broth for all bacterial strains. A stock of resazurin (Sigma-Aldrich) was prepared at 1 mg/mL in sterile PBS. The solution was filter-sterilized and stored at 4 °C in the dark. Three concentrations of resazurin solutions were investigated: 2 μg/mL, 4 μg/mL and 8 μg/mL, and two incubation temperatures: 25 °C and 37 °C. The diluted resazurin solution in PBS was prepared only on the day of the assay. For the assay, firstly biofilm was carefully washed with 200 μL of PBS. Next, 100 μL of diluted resazurin solution was added into each well containing biofilm, along with its respective negative controls (un-inoculated broth, three wells). Microplates were placed in the dark and incubated at 25 °C or 37 °C for 20 min. A multimode microplate reader (Varioskan LUX, Thermo Fisher Scientific) was used to measure the relative fluorescence units (RFU) (λ_Ex_ 530 nm and λ_Em_ 590 nm) after incubation. Readings were repeated at 20-min intervals for up to 80 min. The experiment was performed twice with three replicates. The optimal conditions were chosen based on the analysis of quality parameters (Z’ > 0.50) defined in the assays (see Additional file 1).

### Determination of MBIC and MBEC

Ciprofloxacin hydrochloride (ICN Biomedicals) and linezolid (Sigma-Aldrich) stock solutions were prepared in sterile water to a concentration of 1.6 mg/mL (MBIC assays) and 32 mg/mL and 16 mg/mL for ciprofloxacin and linezolid, respectively (MBEC assays). MBIC and MBEC assays were performed by the broth microdilution method in 96-well flat bottom microplate format adapted from the Clinical & Laboratory Standards Institute (CLSI) guidelines [9, 13].

Briefly, bacterial suspension was diluted with TSG broth to obtain an inoculum of 1 × 10^6^ CFU/mL. Equal volumes of bacterial suspension and antibiotic solution were diluted into TSG broth, mixed together in the plate and incubated for 24 h at 37 °C. Known minimal inhibitory concentrations (MIC) of reference antibiotics were used as positive controls (see Table 2 for details). Ciprofloxacin final concentrations tested ranged from 0.031 μg/mL to 8 μg/mL for the MBIC assays and from 0.625 μg/mL to 160 μg/mL for the MBEC assays. Linezolid final concentrations tested ranged from 0.031 μg/mL to 8 μg/mL for the MBIC assays and from 0.313 μg/mL to 80 μg/mL for the MBEC assays. After incubation, MBIC was defined by performing the optimized resazurin assay (i.e. *Staphylococci* biofilms were assayed using 4 μg/mL of resazurin solution with incubation at 25 °C for 20 min, and *Enterococci* biofilms were assayed using 8 μg/mL of resazurin solution with incubation at 25 °C for 40 min). CFUs were also determined after resazurin-based assay completion, in order to corroborate the results. Biofilm cells were suspended by vigorous pipetting and detached from wells. Cells were quantified by the drop-plate as described above. The MBEC was carried out in a similar manner to the MBIC, however, the *Staphylococci* and *Enterococci* biofilms were initially grown for 24 h and 48 h, respectively, at 37 °C and then treated with antibiotics for 24 h at 37 °C. All experiments were performed twice with three replicates.

**Table 2:** Minimal biofilm inhibitory and eradication concentrations (MBIC and MBEC) determined by resazurin and cell counting methods

Bacterial strain	Antibiotic	MIC^a^ (μg/mL)	MBIC (μg/mL)		MBEC (μg/mL)	
			Resazurin	CFU/mL^b^	Resazurin	CFU/mL^b^
			(decrease in RFU)	(growth inhibition)	(decrease in RFU)	(log_10_ reduction)
*Staphylococcus aureus* ATCC 29213	Ciprofloxacin	0.5	1 (99%)	1 (100%)	> 160 (18%)	> 160 (1.2)
*Staphylococcus aureus* MRSA ATCC 43300	Ciprofloxacin	0.5	1 (99%)	1 (100%)	> 160 (30%)	> 160 (1.7)
*Enterococcus faecalis* ATCC 29212	Ciprofloxacin	1	1 (90%)	1 (100%)	> 160 (35%)	> 160 (4.0)
*Enterococcus faecalis* VRE ATCC 51575	Ciprofloxacin	0.5	1 (99%)	1 (100%)	> 160 (0%)	> 160 (3.3)
*Enterococcus faecium* ATCC 35667	Linezolid	4	2 (96%)	2 (100%)	> 80 (26%)	> 80 (1.5)
*Enterococcus faecium* VRE ATCC 70021	Linezolid	2	2 (99%)	2 (97%)	> 80 (26%)	> 80 (1.8)

Mean of quality parameters of MBIC and MBEC assays were: Z’ = 0.83; S/B = 14.83, SW = 36.40 and Z’ = 0.77; S/B = 21.19 and SW = 17.19, respectively

^a^:Minimal inhibitory concentration previously determined by our group

^b^:For each experiment, initial biofilm cell populations were determined and used for the calculations of growth inhibition and log_10_ reduction

### Data analysis

The data obtained from the different media tested for biofilm growth were evaluated by Analysis of Variance (ANOVA) and then Tukey’s test using SPSS version 25 software (IBM). *P* values of less than 0.05 were regarded as significant.

The resazurin optimization assays were assessed by assay quality parameters typically employed in the development of new screening methods, i.e. Z′, S/B and SW [14]. These parameters were also used for assessing the quality of the data obtained during the determinations of MBIC and MBEC. The Z’ is reflective of both the assay signal dynamic range and the data variation associated with single measurements. The following equations were used: Z*′* = 1 – [(3SDs + 3SDb)/|Xs – Xb|], S/B = Xs / Xb and SW = [Xs – Xb – 3 (SDs + SDb)]/ SDs, where Xs represents the average of the signal obtained from control samples exhibiting maximum signal and SDs the related standard deviation, and Xb and SDb represent the average and standard deviation of the signal obtained from control media wells. The threshold value for Z*′* is 0.5, indicating an excellent performance for the assay [11].

MBIC values were determined as the lowest concentration of antibiotic that displayed biofilm inhibition of > 90% based on RFU and CFU determinations. From MBEC experiments, where an MBEC value could not be determined, the percentage of decrease compared to untreated samples was calculated.

## Results

### Assessment of biofilm biomass and cell numbers

In this study, biofilm production of six bacterial strains (Table 1) on six different media: MHB, TS, TSG, TS2G, BHI, BHIG, after 24 h and 48 h incubation for *Staphylococci* and *Enterococci*, respectively, was evaluated. Two methods frequently used for biofilm studies were applied to assess biofilm production: CV and determination of CFUs.

**Fig. 1:**
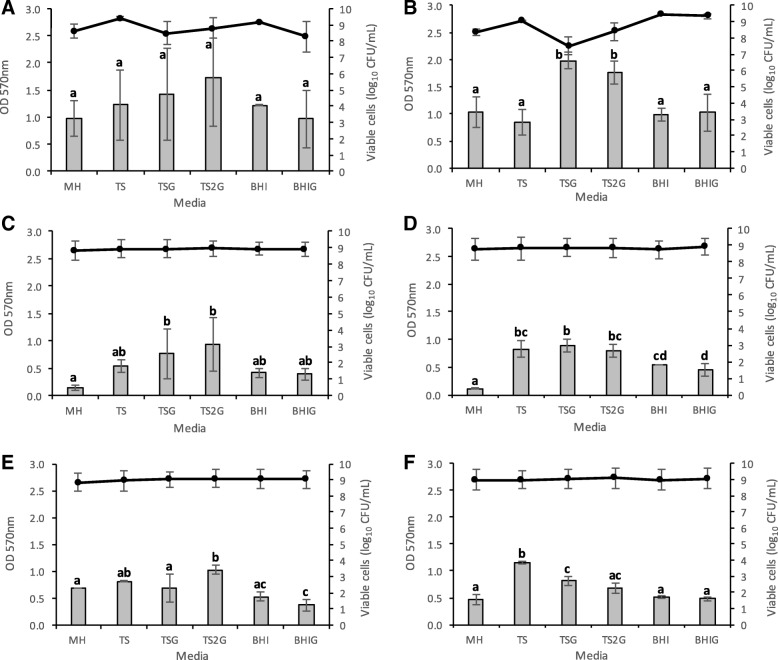
Assessment of biofilm biomass by crystal violet staining and cell enumeration by colony count. Biofilm production analysis through crystal violet staining (OD 570 nm, bars) and cell enumeration (log_10_CFU/mL, lines) of six bacterial strains: (**a**) *Staphylococcus aureus* ATCC 29213, (**b**) *Staphylococcus aureus* MRSA ATCC 43300, (**c**) *Enterococcus faecium* ATCC 35667, (**d**) *Enterococcus faecium* VRE ATCC 700221, (**e**) *Enterococcus faecalis* ATCC 29212 and (**f**) *Enterococcus faecalis* VRE ATCC 51575, incubated in six different media: Mueller Hinton (MH), Tryptic Soy (TS), Tryptic Soy supplemented with 1% glucose (TSG), Tryptic Soy supplemented with 2% glucose (TS2G), Brain Heart Infusion (BHI) and Brain Heart Infusion supplemented with 1% glucose (BHIG). Error bars represent standard deviation of two independent experiments in triplicate. Lowercase letters indicate significant differences amongst media used for biofilm production. Similar letters denote no significant difference (*P* > 0.05)

Figure 1 shows the average results for all conditions tested. Biofilm mass, measured by the absorbance of CV at 570 nm was relatively low for most *Enterococcus* spp. tested in comparison to *Staphylococcus* spp., with values ranging from 0.109 to 1.151, in contrast to 0.849 to 1.984, respectively. Both *E. faecium* strains had the lowest biofilm mass in MHB compared with other tested strains, with an average of 0.149 for *E. faecium* ATCC 35667 (Fig. 1c) and 0.109 for vancomycin resistant *E. faecium* (VRE) ATCC 700221 (Fig. 1d). It was observed that supplementing TS broth with glucose increased the biofilm production of *S. aureus* and *E. faecium* ATCC 35667 strains (Fig. 1a-c), although a significant difference was only observed for the methicillin-resistant *S. aureus* (MRSA) strain (*P* < 0.05) (Fig. 1b). No significant difference was observed between 1 and 2% glucose supplementation (*P* > 0.05), except for *E. faecalis* ATCC 29212 (Fig. 1e). Supplementation of BHI with glucose did not cause any significant difference (P > 0.05) for any of the strains tested (Fig. 1).

Average viable cell counts ranged between 7.5 and 9.4 log_10_ CFU/mL. In general, no differences were observed in the number of viable cells and the different media used for biofilm production. The lowest CFU was observed when MRSA biofilm was grown in TSG medium, although it produced the highest biofilm mass according to the CV assay (Fig. 1b). This suggests that the increased biofilm mass observed is probably due to higher production and/or aggregation of extracellular substances in the biofilm matrix, and not to a direct increase in cell numbers.

### Assessment of metabolic activity of biofilm cells by resazurin

**Fig. 2:**
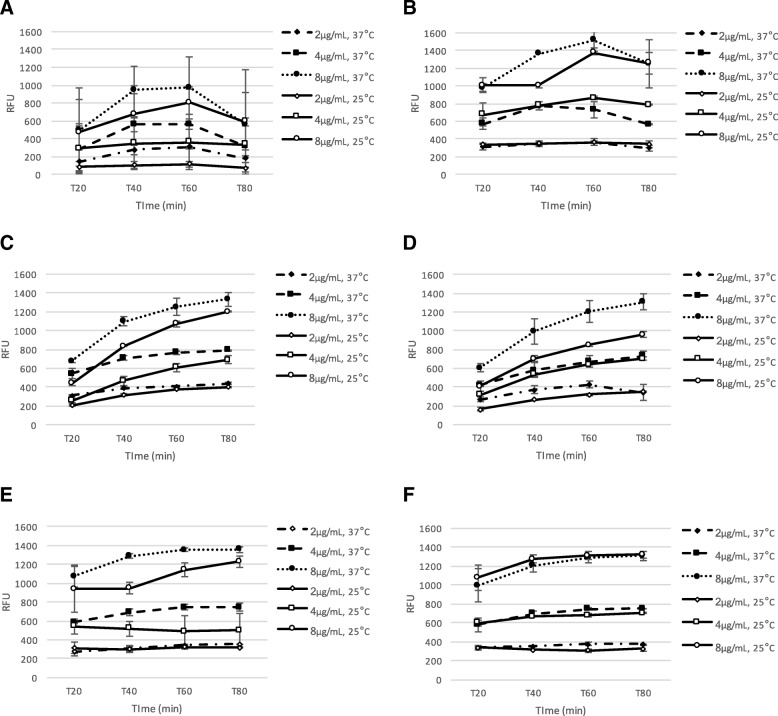
Assessment of metabolic activity of biofilm cells by resazurin. Quantification of biofilm production of six bacterial strains, using three concentrations of resazurin solution (2, 4 and 8 μg/mL): (**a**) *Staphylococcus aureus* ATCC 29213, (**b**) *Staphylococcus aureus* MRSA ATCC 43300, (**c**) *Enterococcus faecium* ATCC 35667, (**d**) *Enterococcus faecium* VRE ATCC 700221, (**e**) *Enterococcus faecalis* ATCC 29212, (**F**) *Enterococcus faecalis* VRE ATCC 51575, incubated at 25 °C and 37 °C. Relative fluorescence measurements were taken at 20, 40, 60 and 80 min

Studies have shown that resazurin viability assay is a good alternative for quantification of biofilms grown in microplates [9, 15]. Evaluation of assay’s performance and the selection of optimal assay conditions were based on the use of typical statistical quality parameters, as described by Zhang et al. [16] and Inglese et al. [11]. Overall, the RFU, which are defined as the arbitrary units in which fluorescence intensity is reported, were higher when using resazurin solution at 8 μg/mL and when plates were incubated at 37 °C (Fig. 2), except for *E. faecalis* VRE ATCC 51575 (Fig. 2e). For both strains of *E. faecalis*, RFU values were still increasing at the maximum incubation time of this study (i.e. 80 min) (Fig. 2c and d). For the other strains, a steady or even lower RFU values were observed at the end of the assays (Fig. 2).

Examination of the results for quality parameters (Additional file 1) revealed that most of the combinations of conditions selected for the different resazurin assays yielded an excellent Z’ value (i.e. ≥ 0.5), with the exception of *S. aureus* ATCC 29213. For this strain, higher variability was observed between replicates. Similar results were obtained in previous experiments when testing different media for biofilm production (Fig. 1a).

The conditions for the resazurin assay selected for further experiments were set as follows: 4 μg/mL and 8 μg/mL, with incubation at 25 °C for 20 min and 40 min for *Staphylococci and Enterococci*, respectively. These were selected based on the quality parameter Z’, as well on previous published data (to facilitate further comparison with other studies [17–19]), when Z’ was satisfactory in more than one combination of parameters.

### MBIC and MBEC determination

Using the previously optimized assays, we assessed the MBIC and MBEC of ciprofloxacin and linezolid for the six bacterial strains. Biofilms were grown in TSG media and resazurin assays were conducted as follows: 4 μg/mL of resazurin with an incubation of 20 min at 25 °C for *Staphylococci* biofilms and 8 μg/mL of resazurin with an incubation of 40 min at 25 °C for *Enterococci* biofilms, based on findings obtained during the assessment of metabolic activity of biofilm cells (Fig. 2).

A limitation of using the resazurin-based assay is the low linear range and detection limit of viable cells (i.e. 10^6^ cells per biofilm, [15]). Thus, conditions leading to higher RFU values were chosen, combined with the quality parameters’ assessment, for an acceptable assay. In order to validate the results obtained with the resazurin-based assay for MBIC and MBEC determinations, CFU counts were also determined using the same samples.

Two strains presented a MBIC value similar to their respective MIC, while *S. aureus* strains and *E. faecalis* VRE ATCC 51575 had a ciprofloxacin MBIC higher than the MIC by 2-fold. *E. faecium* ATCC 35667, showed a 2-fold lower linezolid MBIC than its respective MIC. Although differences were observed for MIC and MBIC of some bacterial strains, these are not significant. MBIC values were similar by two methods employed: resazurin and CFU counts. The quality of the resazurin-based assay for MBIC determination was acceptable, with an average Z’ of 0.83 and S/B and SW higher than 2-fold (Table 2) [11].

The MBEC was defined as the minimal concentration of antibiotic required to reduce biofilm cell numbers below detection limit of the assays used (i.e. 10^6^ CFU/mL for resazurin-based assay and 10^2^ CFU/mL for cell counting method). We also consider that a successful biofilm eradication should be complete, thus there is no bacterial survivors to multiply and restore colonization. Based on these assumptions MBEC was not achieved with the two tested antibiotics even at the highest concentrations.

Evaluation of the percentage of decrease obtained in the MBEC assays showed that the results varied depending on the assay used, i.e. for the resazurin assay RFU values showed a lower decrease in comparison to bacterial quantification method (Table 2). This finding corroborates what has been shown by Peeters et al. [15], elucidating the limitation of the use of the resazurin assays for some applications on biofilm research.

Overall a 3 to 4 log_10_ CFU reduction was achieved by treating biofilm cells of *E. faecalis* ATCC 35667 and *E. faecalis* VRE ATCC 51575 with 160 μg/mL ciprofloxacin, respectively. This reduction corresponded to a decrease in RFU of about 35% and 16%, while for other bacterial biofilms tested, at maximum antibiotic concentrations, only 1.7 log_10_ CFU reduction or even less was observed (Table 2).

## Discussion

Stepanovic et al. [9] reviewed several studies on testing different conditions for *S. aureus* biofilms. Concordant with our results, TS broth supplemented with 1% glucose had the best performance, with an incubation time of 24 h [12, 20]. Some strains of *Staphylococci* have been shown to produce more biofilm in BHI [20, 21].

*Enterococci* biofilm production also differs between different species depending on the media used in the assays. Baldassari et al. [22] and Pilai et al. [23] reported that TS broth supplemented with 1% glucose increased biofilm production of *E. faecalis*, which is similar to our findings. Mohamed et al. [6] compiled some studies on biofilm production by *Enterococci* that suggested that *E. faecalis* produces biofilm more often and stronger than *E. faecium*, independent of the media used in the assays. TS broth supplemented with 0.5% to 1% glucose seems to be a common media used elsewhere for *Enterococcus* spp. biofilm [24–26].

Composition of the medium is probably the most important factor influencing the ability of bacteria to produce biofilm under in vitro conditions. Accordingly, presence of carbohydrate plays an important role in biofilm production amongst Gram-positive bacteria.

Although a clear recommendation for the use of a single medium appropriate for all *Staphylococci* and *Enterococci* when testing biofilms is difficult, our results revealed TS broth supplemented with 1% glucose to be the medium of choice for the clinical laboratory reference strains tested in our study.

Optimal incubation time also differed between bacterial species. *Enterococcus* spp. had a poor biofilm production after a 24 h incubation at 37 °C (data not shown); the incubation time was therefore extended to 48 h. This phenomenon has been observed elsewhere [27], mostly for VRE strains [25].

The development of new anti-biofilm agents is hindered by the lack of a reliable and accurate method for screening of activity (i.e. determination of MBIC and/or MBEC). To determine antibacterial activity, several studies have combined two staining assays for measuring the total biomass and viability of biofilms using CV, CFU and/or resazurin [17, 28, 29–32].

Here, we opted for the resazurin assay to establish the MBIC and MBEC for selected antibiotics. This methodology has some pros and cons as described by Sandberg et al. and Peeters et al. [15, 18]; they noted that optimal resazurin parameters should be established for every tested bacterial strain when using this method as a screening tool. The results in RFU values generated from the assay have also to be interpreted carefully since this method has a lower limit of quantification (method cannot discriminate cell numbers lower than 10^6^ CFU per biofilm). Thus lower numbers of viable cells cannot be measured, requiring a supplemented method, such as CFU counts. Van den Driessche [33] has recently described an optimized resazurin-based quantification for some microbial biofilms lowering the detection limit of 10^3^ CFU. Nevertheless, the resazurin assay is a very easy and reproducible method, ideal for higher throughput in screening, and when used for the determination of MBIC it performs well.

The quality of the resazurin assay was evaluated based on its reproducibility and on three quality parameters: SW, S/B and Z’. To our knowledge, this is the first study to evaluate the resazurin assay for MBIC and MBEC determinations using this approach.

The difficulty in eradicating *S. aureus* biofilms using ciprofloxacin is consistent with earlier data using other antibacterial agents [34, 35]. The selection of ciprofloxacin for our study was based on its current use as a standard positive control for MIC determinations as recommended by CLSI [13]. For the determination of *S. aureus* MBEC, another drug should be selected as a positive control. Still the results presented in this work show the feasibility of a standardized resazurin assay for MBIC and MBEC determinations, followed by an appropriate quality assessment by using statistical parameters. Overall, MBEC requires a complementing method to accurately determine effects on biofilms [28, 36].

In this study, the resazurin assay was used to determine the MBIC and MBEC values. Data obtained reveal that the resazurin assay is an acceptable choice for MBIC determination. The anti-biofilm assays of selected antibiotics were performed under optimized biofilm growth conditions and detection of metabolically active cells for Gram-positive laboratory reference strains. Bridging the gap between results obtained in in vitro to in vivo outcomes is challenging, thus this present study only provided guidelines for screening tools that could be useful in the biofilm research field.

## Conclusions

The clinical relevance of S*taphylococci* and *Enterococci* is related to their ability to form biofilms, which are complex biological structures highly tolerant to antibiotic treatment.

This study shows the importance of media selection for biofilm growth and the optimization of assays concurrent with quality parameter assessment. We have described here a useful and standardized tools for MBIC and MBEC determinations when screening and evaluating compounds with potential anti-biofilm activity. The clinical impact of the data obtained using the optimized methodology has not been evaluated in this study, thus further experiments are required to assess this aspect.

## Additional file

Additional file 1: Quality parameters from resazurin optimization assays for assessing metabolic activity of biofilm cells. This table provides all the quality parameter (Z prime, signal to background and signal window) results calculated for each of the resazurin conditions tested and for the six bacterial strains used in the study. (DOCX 35 kb)

## Abbreviations

ANOVA: Analysis of variance
BHI: Brain heart infusion
BHIG: Brain heart infusion supplemented with 1% glucose
CFU: Colony-forming units
CLSI: Clinical & Laboratory Standards Institute
CV: Crystal violet
*E. faecalis*: *Enterococcus faecalis*
*E. faecium*: *Enterococcus faecium*
EPS: Exopolysaccharide
MBEC: Minimal biofilm eradication concentration
MBIC: Minimal biofilm inhibitory concentration
MHA: Mueller hinton agar
MHB: Mueller hinton broth
MIC: Minimal inhibitory concentration
MRSA: Methicillin-resistant *S. aureus*
OD: Optical density
PBS: Phosphate-buffered saline
RFU: Relative fluorescence units
*S. aureus*: *Staphylococcus aureus*
S/B: Signal to background
SW: Signal window
TS: Tryptic soy
TS2G: Tryptic soy supplemented with 2% glucose
TSG: Tryptic soy supplemented with 1% glucose
VRE: Vancomycin-resistant *Enterococcus*
Z’: Z prime
