# Supplementary material for: Defining conditions for biofilm inhibition and eradication assays for Gram-positive clinical reference strains
Source: BMC Microbiol. 2018 Nov 3;18:173. doi: 10.1186/s12866-018-1321-6 (PMC6215609; doi:10.1186/s12866-018-1321-6)
Supplement: Supplementary file 1 — Quality parameters from resazurin optimization assays for assessing metabolic activity of biofilm cells. This table provides all the quality parameter (Z prime, signal to background and signal window) results calculated for each of the resazurin conditions tested and for the six bacterial strains used in the study. (DOCX 35 kb) [file 12866_2018_1321_MOESM1_ESM.docx]

**Additional File 1 (doc)**

| Bacterial strain | Resazurin concentration (µg/mL) | Temperature (°C) | 25 | | | | | | | | 37 | | | | | | |
| --- | --- | --- | --- | --- | --- | --- | --- | --- | --- | --- | --- | --- | --- | --- | --- | --- | --- |
|  |  | Time (min) | 20 | 40 | | 60 | | 80 | | 20 | | 40 | | | 60 | | 80 |
|  |  | Quality parameters* |  |  | |  | |  | |  | |  | | |  | |  |
| *Staphylococcus aureus ATCC* 29213 | 2 | S/B | 4.32 | | 4.80 | | 5.08 | | 3.64 | | 7.23 | | 12.56 | 12.27 | | 8.25 | |
|  |  | SW | 2.51 | | 2.17 | | 1.72 | | 1.71 | | 4.29 | | 5.38 | 4.38 | | 2.48 | |
|  |  | Z' | -0.22 | | -0.66 | | -1.26 | | -1.33 | | 0.16 | | 0.37 | 0.21 | | -0.38 | |
|  | 4 | S/B | 7.77 | | 8.42 | | 7.98 | | 7.96 | | 7.61 | | 14.80 | 12.81 | | 7.65 | |
|  |  | SW | 38.08 | | 12.51 | | 11.36 | | 10.10 | | 3.12 | | 14.18 | 20.96 | | 22.63 | |
|  |  | Z' | 0.49 | | 0.49 | | 0.00 | | -0.25 | | -1.27 | | 0.74 | 0.83 | | 0.54 | |
|  | 8 | S/B | 7.90 | | 10.42 | | 10.97 | | 9.52 | | 8.63 | | 16.10 | 14.98 | | 9.65 | |
|  |  | SW | 14.98 | | 6.90 | | 3.63 | | 3.69 | | 6.81 | | 8.21 | 11.80 | | 8.34 | |
|  |  | Z' | 0.72 | | 0.51 | | -0.31 | | -3.02 | | 0.20 | | 0.63 | 0.63 | | 0.26 | |
| *Staphylococcus aureus MRSA ATCC* 43300 | 2 | S/B | 14.61 | | 14.59 | | 14.56 | | 15.02 | | 13.46 | | 14.66 | 15.21 | | 11.90 | |
|  |  | SW | 8.95 | | 17.34 | | 8.37 | | 12.28 | | 14.19 | | 11.60 | 10.87 | | 11.43 | |
|  |  | Z' | 0.52 | | 0.78 | | 0.63 | | 0.72 | | 0.77 | | 0.72 | 0.61 | | 0.50 | |
|  | 4 | S/B | 16.99 | | 18.47 | | 20.09 | | 20.01 | | 14.48 | | 20.53 | 19.09 | | 14.15 | |
|  |  | SW | 14.84 | | 21.95 | | 18.73 | | 17.59 | | 27.60 | | 38.43 | 35.36 | | 51.58 | |
|  |  | Z' | 0.79 | | 0.86 | | 0.84 | | 0.82 | | 0.84 | | 0.92 | 0.91 | | 0.94 | |
|  | 8 | S/B | 16.34 | | 18.10 | | 20.04 | | 19.81 | | 16.11 | | 23.92 | 25.88 | | 20.08 | |
|  |  | SW | 24.36 | | 11.66 | | 17.04 | | 16.80 | | 25.85 | | 21.37 | 38.76 | | 33.76 | |
|  |  | Z' | 0.53 | | 0.71 | | 0.81 | | 0.80 | | 0.87 | | 0.82 | 0.83 | | 0.91 | |
| *Enterococcus faecium ATCC* 35667 | 2 | S/B | 9.36 | | 13.66 | | 16.00 | | 16.80 | | 14.09 | | 17.25 | 17.82 | | 19.06 | |
|  |  | SW | 23.56 | | 27.80 | | 36.58 | | 36.12 | | 10.85 | | 18.71 | 19.88 | | 25.69 | |
|  |  | Z' | 0.81 | | 0.88 | | 0.88 | | 0.81 | | 0.72 | | 0.83 | 0.84 | | 0.85 | |
|  | 4 | S/B | 7.24 | | 12.13 | | 14.83 | | 16.69 | | 14.69 | | 18.72 | 20.16 | | 20.90 | |
|  |  | SW | 8.99 | | 11.18 | | 16.42 | | 23.50 | | 27.69 | | 21.53 | 50.15 | | 17.45 | |
|  |  | Z' | 0.62 | | 0.70 | | 0.78 | | 0.83 | | 0.85 | | 0.68 | 0.64 | | 0.70 | |
|  | 8 | S/B | 7.72 | | 13.69 | | 17.22 | | 19.23 | | 11.23 | | 17.43 | 19.78 | | 20.94 | |
|  |  | SW | 7.58 | | 12.75 | | 16.56 | | 18.36 | | 9.23 | | 29.38 | 39.07 | | 38.98 | |
|  |  | Z' | 0.44 | | 0.70 | | 0.77 | | 0.83 | | 0.66 | | 0.90 | 0.92 | | 0.92 | |
| *Enterococcus faecium VRE ATCC* 70021 | 2 | S/B | 7.80 | | 11.88 | | 14.14 | | 15.67 | | 11.91 | | 15.88 | 17.83 | | 19.30 | |
|  |  | SW | 4.87 | | 6.59 | | 10.58 | | 12.58 | | 20.07 | | 23.74 | 29.05 | | 45.32 | |
|  |  | Z' | 0.29 | | 0.53 | | 0.66 | | 0.70 | | 0.77 | | 0.83 | 0.86 | | 0.89 | |
|  | 4 | S/B | 9.00 | | 14.57 | | 17.15 | | 18.77 | | 11.57 | | 15.67 | 17.92 | | 19.54 | |
|  |  | SW | 5.93 | | 10.39 | | 11.07 | | 11.59 | | 6.37 | | 9.57 | 9.09 | | 7.83 | |
|  |  | Z' | 0.45 | | 0.63 | | 0.71 | | 0.72 | | 0.49 | | 0.67 | 0.65 | | 0.60 | |
|  | 8 | S/B | 7.27 | | 11.71 | | 13.85 | | 15.63 | | 10.11 | | 15.92 | 18.98 | | 20.88 | |
|  |  | SW | 6.12 | | 10.68 | | 17.51 | | 37.29 | | 17.62 | | 46.69 | 81.64 | | 42.33 | |
|  |  | Z' | 0.50 | | 0.70 | | 0.82 | | 0.85 | | 0.82 | | 0.90 | 0.95 | | 0.92 | |
| *Enterococcus faecalis ATCC* 29212 | 2 | S/B | 15.44 | | 11.74 | | 12.07 | | 14.94 | | 12.66 | | 13.16 | 17.20 | | 17.04 | |
|  |  | SW | 340.14 | | 40.88 | | 8.52 | | 31.99 | | 7.92 | | 4.59 | 13.63 | | 11.30 | |
|  |  | Z' | 0.96 | | 0.87 | | 0.63 | | 0.75 | | 0.47 | | 0.31 | 0.68 | | 0.65 | |
|  | 4 | S/B | 16.04 | | 12.43 | | 12.66 | | 13.26 | | 17.65 | | 19.61 | 20.40 | | 20.17 | |
|  |  | SW | 47.47 | | 41.28 | | 81.14 | | 23.78 | | 28.20 | | 74.69 | 74.85 | | 78.22 | |
|  |  | Z' | 0.93 | | 0,91 | | 0.92 | | 0.52 | | 0.88 | | 0.95 | 0.96 | | 0.96 | |
|  | 8 | S/B | 19.03 | | 15.97 | | 17.73 | | 19.56 | | 18.25 | | 19.62 | 19.86 | | 19.97 | |
|  |  | SW | 83.80 | | 22.52 | | 18.64 | | 41.41 | | 25.95 | | 26.51 | 29.11 | | 25.27 | |
|  |  | Z' | 0.95 | | 0.86 | | 0.69 | | 0.92 | | 0.87 | | 0.88 | 0.89 | | 0.87 | |
| *Enterococcus faecalis VRE ATCC* 51575 | 2 | S/B | 14.12 | | 13.08 | | 12.72 | | 13.23 | | 15.30 | | 16.68 | 17.51 | | 16.77 | |
|  |  | SW | 22.56 | | 44.08 | | 131.94 | | 29.18 | | 18.21 | | 18.46 | 15.91 | | 14.72 | |
|  |  | Z' | 0.73 | | 0.80 | | 0.89 | | 0.87 | | 0.73 | | 0.80 | 0.79 | | 0.78 | |
|  | 4 | S/B | 15.23 | | 16.85 | | 16.89 | | 17.35 | | 15.09 | | 17.83 | 18.83 | | 18.99 | |
|  |  | SW | 108.82 | | 63.29 | | 53.52 | | 127.56 | | 18.93 | | 59.82 | 73.40 | | 106.44 | |
|  |  | Z' | 0.94 | | 0.90 | | 0.91 | | 0.96 | | 0.84 | | 0.95 | 0.95 | | 0.97 | |
|  | 8 | S/B | 16.20 | | 19.01 | | 19.29 | | 19.56 | | 19.03 | | 20.76 | 21.63 | | 21.66 | |
|  |  | SW | 44.47 | | 43.79 | | 41.45 | | 42.86 | | 22.11 | | 32.45 | 55.57 | | 109.47 | |
|  |  | Z' | 0.88 | | 0.92 | | 0.92 | | 0.90 | | 0.81 | | 0.89 | 0.94 | | 0.96 | |

*: Quality parameters presented in the table are the average values for two experiments in triplicate. S/B: Signal to background, SW: Signal window, Z’: Z prime. Acceptable assay performance is defined by S/B and SW > 2- fold and Z’ > 0.5.
